# Supplementary material for: Protein Recovery of Tra Catfish (Pangasius hypophthalmus) Protein-Rich Side Streams by the pH-Shift Method
Source: Foods. 2022 May 24;11(11):1531. doi: 10.3390/foods11111531 (PMC9180071; doi:10.3390/foods11111531)
Supplement: Supplementary file 1 [file foods-11-01531-s001.zip › foods-1725984-supplementary.pdf]

Table S1. List of abbreviations

Abbreviation	Meaning
ACO	Abdominal cut-offs
ACO-FPI	Fish protein isolate produced from the abdominal cut-off
DM-FPI	Fish protein isolate produced from the dark muscle
HBB	Head and backbone blend
HBB-FPI	Fish protein isolate produced from the head and backbone blend
FPI	Fish protein isolate
FPI-DMR	Dry matter recovery of the fish protein isolate compared to the initial raw material
FPI-PR	Protein recovery of the fish protein isolate compared to the initial raw material
LC-MS	Liquid chromatography-mass spectrometry
PES	Protein extraction solution
PER	Protein extractable recovery
PI	Isoelectric protein point
SDS-PAGE	Sodium dodecyl sulfate-Polyacrylamide gel electrophoresis
